# Mechanism of Bonding Reactive Dyes with Copolymer (chloromethyl)oxirane-1H-imidazole cationised Cellulose

**DOI:** 10.3390/ma15134664

**Published:** 2022-07-02

**Authors:** Stanisław Pruś, Piotr Kulpiński, Edyta Matyjas-Zgondek, Krzysztof Wojciechowski

**Affiliations:** 1 Department of Mechanical Engineering, Informatics and Chemistry of Polymer Materials, Łódź University of Technology, ul. Żeromskiego 116, 90-924 Łódź, Poland; piotr.kulpinski@p.lodz.pl (P.K.); edyta.matyjas-zgondek@p.lodz.pl (E.M.-Z.); 2 Institute of Environmental Engineering and Building Installations, Łódź University of Technology, Al. Politechniki 6, 90-924 Łódź, Poland; krzwojc@p.lodz.pl

**Keywords:** cationised cotton, reactive dyes, mechanism bonding, Texamin ECE New, molecular mechanics MM+, quantum-chemical calculations PM3

## Abstract

Introducing the cellulose chain cationic groups in the modification process completely changes the charge on the cotton surface from negative to partially or totally positive. That allows the electrostatic attraction and simultaneous exhaustion and fixation of reactive dyes. This reaction can be carried out without salt and alkali at room temperature. Similarly, the reaction between reactive dye and an alone copolymer ([IME]^+^Cl^−^) with TLC chromatography was confirmed. The analysis with the use of particle optimisation with MM+ molecular mechanics and quantum-chemical calculations PM3 by the method of all valence orbitals confirmed the experimental results of the high activity of the nucleophile formed on the hydroxyl group in the chain of a modifier. It was found and experimentally confirmed that the reactive dyes during the dyeing process of the cotton cationised with copolymer (chloromethyl)oxirane -1H-imidazole ([IME]^+^Cl^−^) create covalent bonds due to a reaction with the hydroxyl group located in the modification agent instead of with the hydroxyl group in the glucopiranose ring. Although the dyeing takes place in very mild conditions, a high degree of setting is achieved, comparable to conventional methods.

## 1. Introduction

Modification of cellulose fibres by cationisation is one of the most frequently described methods that reduces the enormous amount of electrolytes and alkali emitted to the environment in the dyeing process with reactive dyes. In addition, 3-chloro-2-hydroxypropyl- trimethylammonium chloride ([CHPTA]^+^Cl^−^) is the most commonly used cationic modifier due to its low cost and easy chemical reaction with cellulose. Its downside is a lack of substantivity in relation to fibres, which makes it impossible to use it in exhaustion methods. Currently, however, beyond this limitation, there are also other problems. The first is the unpleasant fishy smell that sometimes remains in the material after cationisation as a result of the cleavage of trimethylamine under strongly alkaline conditions [1]. The second is related to security categories. Currently, [CHPTA]^+^Cl^−^ is classified by the EURA (European Union Risk Assessment) as a carcinogen category No. 3 [2]. Other problems concern levelness, penetration and ring dyeing [3]. Perhaps for these reasons, many scientists have turned their attention to cationic cellulose modifiers with a linear or branched polymer structure with medium substantivity to cellulose. Various types of polymers—polyepichlorohydrin dimethylamine, polyepichlorohydrin resin, polyamide epichlorohydrin type polymers, poly(4-vinylpyridine)quaternary ammonium compounds, poly(vinylamine chloride), dendritic polymers, cationic diblock copolymers, chitosan and its derivatives—were presented in reviews [4,5,6,7,8]. In addition, cationic Poly(St-BA-VBT) nanospheres [6], starch and starch derivatives [9] were described in the literature. All of the above-mentioned cationic compounds may be used for cellulose cationisation.

There is still much expectation from the dyeing industry for cationised cellulose that allows obtaining dyeings with good durability in the most ecological conditions. A list of cationic cotton suppliers containing manufacturers from seven countries, mostly from the USA and China, has been published [10]. They offer cationised fibres, yarn, and knitted and woven materials. However, there is no information about cationic modifiers used for this production. One of the modifiers that could potentially solve or strongly reduce most of these problems is copolymer (chloromethyl)oxirane and 1H-imidazole ([IME]^+^Cl^−^) with the commercial name Texamin ECE New, introduced to the market by Inotex spol. s.r.o. (Dvůr Králové nad Labem, Czech Republic) [11]. It reacts with cellulose hydroxyl groups at the same chemistry as [CHPTA]^+^Cl^−^.

[IME]^+^Cl^−^ is a polyheterocyclic cationic liquid copolymer compound. The producer recommends it as a cationising agent to improve cellulose fibres’ dyeability with reactive dyes, resulting in an increasing dyestuff substantivity and lowering in environmental impact [12,13]. It is suitable for processes with reduced salt concentrations, enables one-bath applications for special dyeing effects, and, moreover, is suitable for bath and impregnation processing. [IME]^+^Cl^−^ cationised cotton is also recommended for dyeing with natural dyes [14], forming composites [15], for membrane filtration processes [16] and for preparing innovative UV barrier materials [17]. [IME]^+^Cl^−^ has a GreenScreen Certified™ silver certificate, which prohibits the use of any chemical of high concern listed on globally recognised chemical hazard lists as defined by the GreenScreen List Translator Actio [18]. [IME]^+^Cl^−^ is exported by Inotex spol. s.r.o. to India, Netherlands, etc. [19,20].

Due to the fact that [IME]^+^Cl^−^ has significant market potential, we decided to conduct some research on this product. This work aimed to establish the chemical structure of [IME]^+^Cl^−^ (Texamin ECE New) as well as the reaction mechanism of bonding the reactive dyes with [IME]^+^Cl^−^ cationised cotton. The most environmentally friendly conditions for the dyeing of cationised cellulose, i.e., room temperature and a bath without electrolytes and alkalis, were used. It was assumed that the reaction mechanism of dyeing in those conditions would be similar to that previously discovered when dyeing cationised cellulose with CHPTAC [21]. Five reactive dyes with different reactive groups were selected for the experimental studies. In order to confirm the formation of the covalent bond due to the reaction of the reactive dye with the hydroxyl group of the modifier, methods of extraction with dimethylformamide (DMF) and electron-density analysis were used. The thin layer chromatography method was also applied to analyse the formation of a chemical linkage between the reactive dye and the hydroxyl group belonging to the modifier.

## 2. Materials and Methods

### 2.1. Materials

After alkali scouring and bleaching pre-treatment, the plain cotton fabric with a surface weight of 180 g/m^2^ was used. Cationising agent [IME]^+^Cl^−^ (Texamin ECE New) was purchased from Inotex Czech Republic. Reactive dyes: RR 24:1 from Boruta-Zachem Poland, RB 160 from Kalpactive India, RR 221 from Kisco South Korea, RR 274 from Swisscolor Poland and RB 19 from Biliński Factory Poland were purchased, respectively. Acid dye AB 62 was purchased from Yorkshire Group, Germany. All dyes were applied without further purification. Polyelectrolyte standard solutions: PES-Na (MW 21.800 g/mol) and poly-DADMAC (MW 107.000 g/mol), were purchased from BTG Instruments AB Sweden. Tanaterge Advance (non-ionic detergent) was purchased from Tanachem, Poland. Other chemicals and solvents were used at laboratory grade purity. Ugolini Redkrome–model RED P (Schio VI, Vicenza, Italy) laboratory dyeing machine, heated by infrared ray radiators and equipped with 150/400/5000 mL cups, was used for cationising and dyeing cotton fabric samples. Muetek PCD 03 pH Particle Charge Detector (Muetek GmbH, München, Germany) was used to measure potential and determine the value of the specific surface on the surface of cotton samples. Datacolor 850 spectrofotometer (Datacolor, Lawrenceville, NJ, USA) was used for instrumental colour measurements. Chemical structures and data of modification agents and dyes are shown in Table 1 and Table 2.

### 2.2. Characteristics of [IME]^+^Cl^−^

**Figure 1:**
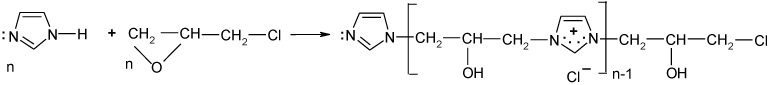
Proposed reaction of the synthesis [IME]^+^Cl^−^.

According to the safety data sheet [11], [IME]^+^Cl^−^ is an adduct of 1H-imidazole and (chloromethyl)-oxirane. The copolymerisation of these products should lead to the copolymer having the following general chemical structure (Figure 1):

Commercial-grade quality [IME]^+^Cl^−^ used for the cationisation of cellulose was analysed for its nitrogen content, cationicity and reactivity values, copolymerisation degree and macromolecular weight.

### 2.3. Cationisation of Cellulose with [IME]^+^Cl^−^

**Figure 2:**
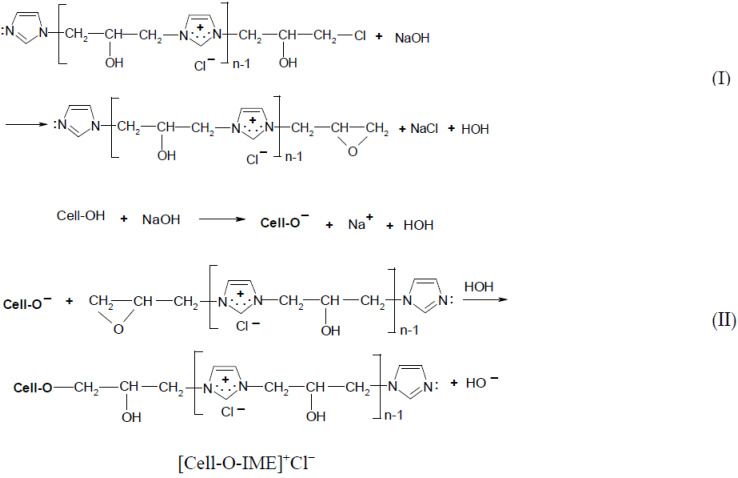
Two-step reaction of cellulose cationisation with [IME]^+^Cl^−l^ (**I**) step = epoxy-form of [IME]^+^Cl^−^ and celulosan anion formation. (**II**) step = covalent bond formed of [IME]^+^Cl^−^ with cellulose.

**Figure 3:**
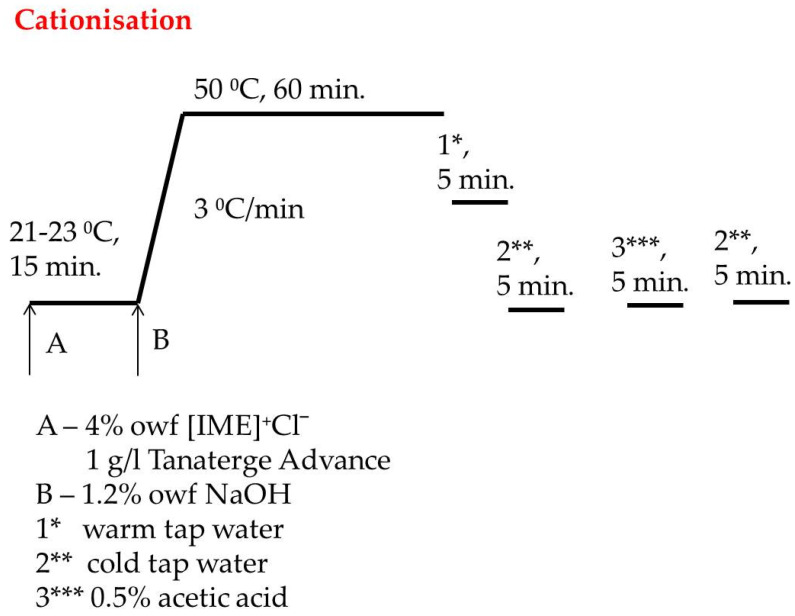
Diagram of catonisation process of cellulose with [IME]^+^Cl^−^.

The cationisation of cellulose was carried out in accordance with the reaction scheme shown below (Figure 2):

A total of 270 g of cotton material was introduced to Ugolini apparatus with 2650 mL bath containing 4% owf [IME]^+^Cl^−^ and 1 g/L Tanaterge Advance and rotated right/left 40 for 15 min at room temperature. Next, 1.2% owf of NaOH dissolved in 50 mL distilled water was added to the apparatus and temperature was increased from 3 °C/min to 50 °C and heating was continued by 60 min. After that, the bath was dropped, and the cationised cotton was rinsed in warm and cold tap water to obtain pH neutrality. Next, the material was acidified with acetic acid 1 g/L to pH 6–7 and finally rinsed again with cold water to neutral pH, then dried at room temperature. The cationisation process is shown in Figure 3.

### 2.4. Eco-Friendly Dyeing of [Cell-O-IME]^+^Cl^−^

**Figure 4:**
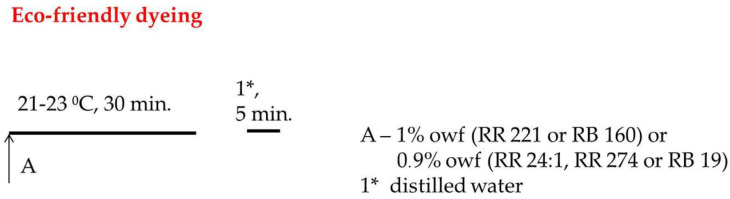
Diagram of dyeing process of cellulose cationised with [IME]^+^Cl^−^.

[Cell-O-IME]^+^Cl^−^ samples were dyed at the following conditions: LR 1:20, temp. 22 ± 1 °C, distilled water and pH neutral with 1% owf (RR 221 or RB 160) and 0.9% owf (RR 24:1, RR 274, or RB 19), respectively, time 30 min, rinsed with cold distilled water to remove not fixed dye and dried at room temperature. For comparing the same conditions, non-cationised cotton samples were dyed and rinsed after dyeing. The temperature course of the dyeing processes is shown in Figure 4.

### 2.5. Characterisation Methods

#### 2.5.1. Nitrogen Content

The values of *N* (eq/g) for the commercial [IME]^+^Cl^−^ and the cotton samples before and after modification were determined by the classical Kjeldahl method described in detail elsewhere [23,24] and calculated according to Equation (1):

(1) $$N=\frac{vc}{14m}\;\;$$

where:

v—mL of hydrochloric acid used for titration;
*c*—concentration of hydrochloric acid 0.1 M [mol/L];
*m*—weight of the sample for analysis [g];
14—atomic mass for Nitrogen.

#### 2.5.2. Specific Charge Measurement

The charge on the surface of cellulose fibres was measured according to the previously developed recipes [23,24]. The value of the specific charge was calculated according to Equation (2):

(2) $$Q_{surf=\frac{\left(v_{0}-v_{1}\right)cv_{c}}{mv_{a}}}^{+}\;$$

where:

v_0_—mL of polyelectrolyte PES-Na for titration of 10 mL polyelectrolyte poly-DADMAC (blind test);
v_1_—mL of polyelectrolyte PES-Na for titration of 10 mL of filtrate after treatment;
*c*—polyelectrolyte concentration of poly-DADMAC;
v_c_—mL of polyelectrolyte poly-DADMAC used for treatment;
v_a_—mL of filtrate used for titration;
*m*—test sample weight [g].

#### 2.5.3. Cationicity

To determine the [IME]^+^Cl^−^ cationicity, 1 g of commercial product was dissolved with 25 × 10^3^ mL of distilled water. An amount equal to 10 mL of the [IME]^+^Cl^−^ solution was placed in a measuring cell of the Muetek PCD 03 pH apparatus, and the piston vibration motion was activated. After stabilising the potential, the solution was titrated with 0.0001 N PES-Na anionic polyelectrolyte to a stable 0 mV potential. The cationicity value was calculated according to Equation (3):

(3) $$Q_{{\left[IME\right]}^{+}Cl^{-}}^{+}=\frac{v_{1}c\;25\times 10^{3}}{mv_{a}}$$

where:

$v_{1}$—mL of polyelectrolyte PES-Na for titration of 10 mL of [IME]^+^Cl^−^ solution;
*C*—concentration of poly-DADMAC polyelectrolyte (eq/L);
$v_{a}$—mL of the [IME]^+^Cl^−^ solution used for titration;
*m*—test sample weight [g].

#### 2.5.4. Reactivity

**Figure 5:**
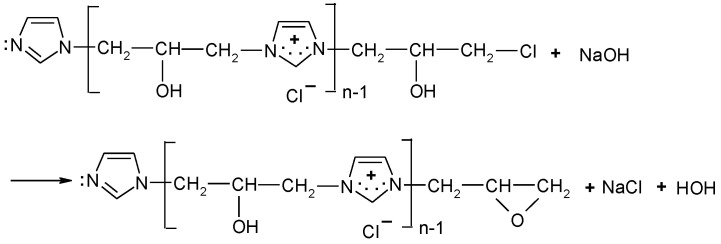
Scheme for creating a reactive form of [IME]^+^Cl^−^.

Reactivity of [IME]^+^Cl^−^ ($R_{{\left[IME\right]}^{+}Cl^{-}\;})$ corresponds to the value of copolymer macromolecules that can react with the hydroxyl group of cellulose in the cationisation process. The amount of NaOH consumed corresponds to the amount of hydrogen chloride released in the epoxide formation according to the reaction (Figure 5):

A total of 1 g of [IME]^+^Cl^−^ commercial product and 40 mL 0.1 N NaOH were heated and stirred in a conical flask with a magnetic stirrer to 60 °C and, after 30 min, cooled to room temperature, then moved to the 50 mL volumetric flask. Then, 10 mL of analysed solution was placed in the 250 mL conical flask, 50 mL of distilled water was added, and next titrated with 0.1 N HCl at phenolphthalein indicator. $R_{{\left[IME\right]}^{+}Cl^{-}}$ was calculated using the following Equation (4):

(4) $$R_{{\left[IME\right]}^{+}Cl^{-}\;}=\frac{\left(v_{o}-v_{1}\right)0.1}{m_{{\left[IME\right]}^{+}Cl^{-}}}\;$$

where:

$v_{o}$—mL of hydrochloric acid used for titration blind sample of 0.1 N NaOH;
$v_{1}$—mL of hydrochloric acid used for titration analysed sample;
$m_{{\left[IME\right]}^{+}Cl^{-}}$—test sample weight [g].

#### 2.5.5. Copolymerisation Degree and Macromolecular Weight of [IME]^+^Cl^−^

Copolymerisation degree and macromolecular weight of [IME]^+^Cl^−^ were calculated according to Equations (5) and (6):

(5) $$DP_{{\left[IME\right]}^{+}Cl^{-}\;}=\frac{0.5\;N}{R_{{\left[IME\right]}^{+}Cl^{-}\;}}\;$$

(6) $$M_{w{\left[IME\right]}^{+}Cl^{-}}=DP_{{\left[IME\right]}^{+}Cl^{-}\;}\times 160.6\;$$

where:

$R_{{\left[IME\right]}^{+}Cl^{-}\;}$—reactivity of [IME]^+^Cl^−^;
$DP_{{\left[IME\right]}^{+}Cl^{-}\;}$—copolymerisation degree of [IME]^+^Cl^−^;
0.5 *N*—number of imidazole molecules in 1 g [IME]^+^Cl^−^;
$M_{w{\left[IME\right]}^{+}Cl^{-}}$—weight of 1 macromolecule [IME]^+^Cl^−^;
160.6—molecular weight of 1 mer of copolymer macromolecule [IME]^+^Cl^−^.

### 2.6. Characterisation of Reaction between Reactive Dye and Modification Agent

Very strong nucleophilicity and reactivity of hydroxyl groups in [IME]^+^Cl^−^ copolymer chain was checked by direct reaction of modification agent with reactive dyes in water at room temperature. In these experiments, the 1% of water solution of [IME]^+^Cl^−^ at room temperature was dropped to the 1% of water solution of reactive dyes. The same test was performed for acid dye AB 62. Precipitated solids were analysed with the TLC method. Pre-coated plastic sheets of Polygram^®^ Sil/G/UV_254_ (Macherey-Nagel + CO, Düren, Germany) were used for chromatography of reactive dyes. As eluent mixture n-propanol:ethylacetate:water = 6:1:3, respectively, was used.

### 2.7. Resistancy for DMF Extraction of Dyed Cationised Cellulose

The resistance to DMF extraction was performed according to the procedure described elsewhere [21]. The colour strength of dyed non-cationised and cationised cotton samples before and after treatment with DMF were determined based on the evaluation of colourimetric measurements using a Datacolor 850 spectrophotometer. The values of resistance of the reactive dyes on dyed cotton samples for DMF extraction were calculated using the following Equation (7):

(7) $$R_{DMF}=\frac{{\left(\frac{K}{S}\right)}_{1}}{{\left(\frac{K}{S}\right)}_{0}}100\;$$

where:

${\left(\frac{K}{S}\right)}_{0}$—value measured after dyeing; and $\;{\left(\frac{K}{S}\right)}_{1}$—value measured after extraction with DMF.

### 2.8. FT-IR Analysis

The chemical structure of [IME]^+^Cl^−^ and cellulose before and after cationisation was analysed by FT-IR spectroscopy in the range 500–4000 cm^−1^ in the Infrared Spectrometer NICOLET 380 (Thermo Fisher Scientific, Waltham, MA, USA). The sample of [IME]^+^Cl^−^ for analysis was prepared by long-term drying at 30–40 °C under a vacuum to remove water. Before analysis, the samples of [IME]^+^Cl^−^, non-cationised and cationised cotton were powdered and mixed with KBr and next pressed into tablet form.

### 2.9. Density Electron Calculations

The structures of the analysed molecules in the ground state were optimised with the use of molecular mechanics MM+ (option with charge analysis) applying Hyperchem v.8.06 programme (Hypercube, Inc., Gainesville, FL, USA) and next analysed with the semi-empirical method of quantum-chemical calculations PM3 [25], taking into account the length of all bonds, angles between them and torsion angles from following the eigenvector procedure (convergence criterion 0.02 kcal/mol).

## 3. Results and Discussion

### 3.1. Characterisation of Copolymer [IME]^+^Cl^−^

The analysis of nitrogen content, cationicity and reactivity of [IME]^+^Cl^−^ presented in Table 3 allows defining and confirming the most likely chemical structure, the average copolymerisation degree and its macromolecular weight.

**Figure 6:**
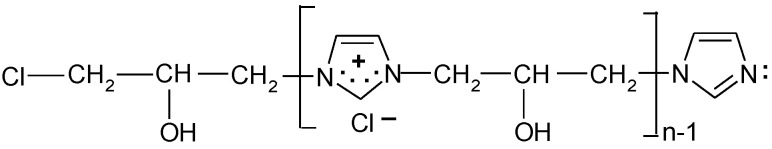
Proposed chemical structure of [IME]^+^Cl^−^ (where: n = 5).

**Figure 7:**
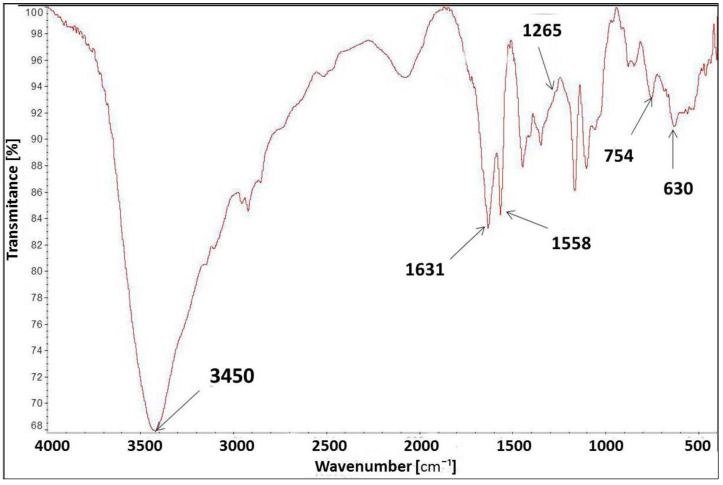
FT-IR spectrum for [IME]^+^Cl^−^.

The average copolymerisation degree and the macromolecule weight of the [IME]^+^Cl^−^ copolymer were calculated according to Equations (5) and (6), and they were 5.01 and 804.6 g, respectively. Based on the obtained data, the structural formula of [IME]^+^Cl^−^ can be drawn as shown in Figure 6:

The FT-IR spectrum of [IME]^+^Cl (Figure 7) showed a characteristic peak at 3450 cm^−1^ for the –OH stretching vibration group. The peak at 1631 cm^−1^ corresponds to the vibration bond present in the imidazole ring for C=C. The peaks at 1558 cm^−1^ and 754 cm^−1^ were assigned to the stretching vibration of the C=N group and the deformation vibration of the imidazole ring, respectively. The band at 630 cm^−1^ was typical of the C-Cl stretching vibration in the CH_2_-Cl group, and the peak at 1265 cm^−1^ was also indicated by this group. The above analysis confirmed the proposed chemical structure of [IME]^+^Cl^−^.

[IME]^+^Cl^−^, being a small length chain cationic copolymer, has medium substantivity to cellulose. The cationic charge in this compound is dislocated in each mer on the whole molecule of Imidazole and has a strong influence on the adjacent hydroxyl group for its acidity and dissociation rate.

### 3.2. Characterisation of [Cell-O-IME]^+^Cl^−^

Cationisation efficiency can be calculated using data from Table 3 and Table 4. Cationisation of cellulose was performed with [IME]^+^Cl^−^ 4% owf, which corresponds to a value of 206.4 × 10^−6^ eq of nitrogen. After cationisation, 101 × 10^−6^ eq of nitrogen was found on the fibres, which corresponds to an approx. 50% yield.

where:
Δ*N*—difference in nitrogen content between cationised and uncationised samples,
$\Delta Q_{surf}^{+}$—difference in specific surface charge between cationised and uncationised samples.

**Figure 8:**
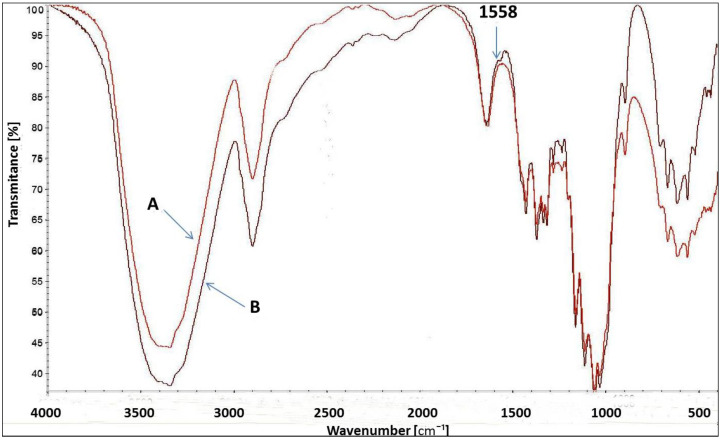
FT-IR spectrum of Cell-OH (A) and [Cell-O-IME]^+^Cl^−^ (B)-(1558 cm^−1^—specific wavenumber. for C=N bond in imidazole ring).

Figure 8 presents the FT-IR spectrum for uncationised and cationised cellulose with [IME]^+^Cl^−^. Both spectra are very similar to each other. However, a slight change in the spectrum of cationised cellulose with wave number 1558 cm^−1^ corresponds to vibration stretching of the C=N bond present in the Imidazole ring belonging to the cationising agent. Such a small difference is due to the low content of the modifier in relation to the weight of cellulose fibres. In the cationisation process, a 4% owf modifier was used. Due to ca. 50% of its fixation, this value dropped to approx. 2%. Such amounts are difficult to identify in the FT-IR spectrum. However, measurements of the amount of nitrogen and the positive charges after cationisation confirmed this modification more clearly.

### 3.3. Characterisation of [Cell-O-IME]^+^Cl^−^ Dyeing

**Figure 9:**
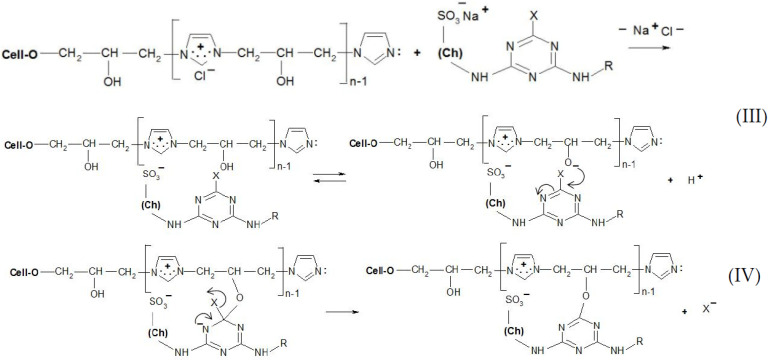
Proposed mechanism of the dyeing [Cell-O-IME]^+^Cl^−^ with triazine dyes: (**III**) ionic formation bonds on the fibre and dissociation of a hydroxyl group in ionic pair: cationised cotton + reactive dye on the fibre, (**IV**) substitution reaction with formation of a covalent bond between reactive dye and nucleophile of the hydroksyl group where: Ch—chromophore of RR 24:1, RB 160, RR 221 and RR 274, R—different chemical substituents in the chemical structure above dyes X—nicotinic acid for RR 221, halogen for the other dyes.

**Figure 10:**
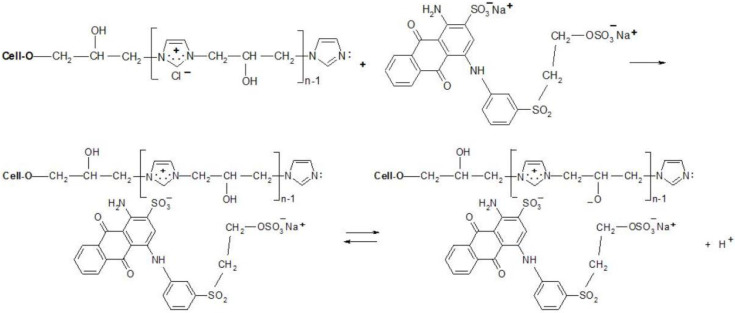

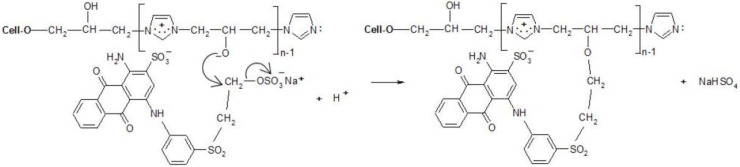
Proposed mechanism of dyeing [Cell-O-IME]^+^Cl^−^ with RB 19 (mono-VS reactive dye).

Thanks to the attractive positive charges on the fibre, the reactive dyes were absorbed quickly from the bath-formed ionic bonds (Figure 9 and Figure 10), having two possible ways of the reaction with [Cell-O-IME]^+^Cl^−^:

(1) Create a covalent bond with one of the hydroxyl groups in the chain of the copolymer by nucleophilic substitution or addition;

(2) React (only for chlorotriazine reactive dyes) with a lone pair on the nitrogen atom of [Cell-O-IME]^+^Cl^−^ to form the quaternary compounds [26]. Such compounds are very reactive and react quickly with the dissociated hydroxyl group in the modifier chain to form a covalent bond.

The reaction mechanisms for selected reactive dyes with cationised cellulose were proposed (Figure 9 and Figure 10):

### 3.4. Evaluation and Confirmation of the Type of Binding between the Reactive Dye and Cationised Cellulose

The research was aimed at confirming the hypothesis that reactive dyes in eco-friendly dyeing conditions can form a covalent bond due to a reaction with the hydroxyl group of the cationic modifier [IME]^+^Cl^−^ instead of with the hydroxyl group of the glucopyranose ring. The results of colour fastness to extraction in boiling DMF confirmed the formation of a covalent bond. Strong nucleophilicity of the hydroxyl groups in the cationic modifier was confirmed by direct reaction with reactive dyes in a neutral aqueous medium. The analysis of electron densities on oxygen and nitrogen atoms in model compounds also confirms the hypothesis of the site of covalent-bond formation.

#### 3.4.1. Resistance of Dyed [Cell-O-IME]^+^Cl^−^ for Extraction with DMF

All obtained experimental data presented in Table 5 show that the samples of cationised cotton dyed with reactive dyes in eco-friendly conditions are fast for extraction with boiling DMF in contrast to the samples of non-cationised cellulose. It confirmed covalent bonds between the reactive group of the dye and the hydroxyl group in the modifier chain. The *R_DMF_* presented in Table 5 is also on the level of these values for conventional dyeing methods with dyes described in the research of [21]. Obtained results are comparable and even better than on cellulose cationised with CHPTAC and dyed in the same eco-friendly conditions.

The *R_DMF_* value for RR 274 of higher than 100% is probably a result of the aggregation of the dye molecule on the surface of cationic cellulose fibres. Dye aggregates can be broken during DMF extraction leading to an increase in colour strength and an increase in the degree of bonding, up to 100%. It can be noted that this phenomenon is more intense for Cell-O-[HPTA]^+^Cl^−^ (our earlier study [21]) than for [Cell-O-IME]^+^Cl^−^.

#### 3.4.2. Reaction between Reactive Dyes and Modification Agent

When, to the 10 mL of 1% water solution of reactive dye, 1% water solution of [IME]^+^Cl^−^ was added, insoluble solid precipitate immediately formed. Initially, a small quantity of insoluble solid with a bigger colourful halo on filter paper was observed. Next, portions of the 1% solution [IME]^+^Cl^−^ formed a total colourless halo, and the filtrate was transparent. The precipitated solid was rinsed with water to obtain an entirely colourless bath and then filtered and dried at room temperature. The same reaction with 1% water solution of acid dye AB 62 was observed for comparison.

**Figure 11:**
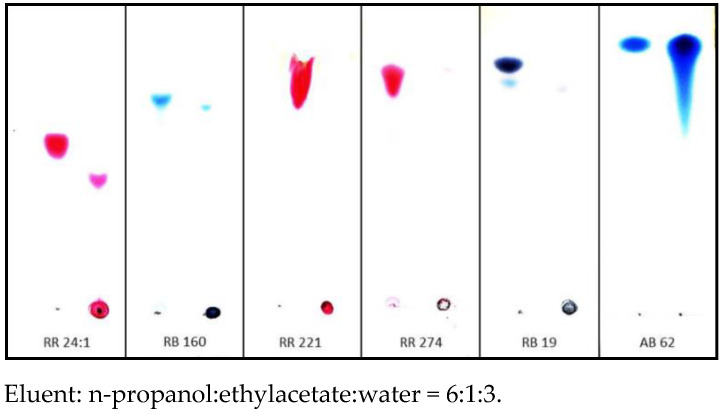
TLC chromatography coloured compounds obtained in reactions between reactive dyes and acid dye with [IME]^+^Cl^−^ (left side—analysed dye, right side—the reaction product of dye with [IME]^+^Cl^−^).

Dry reaction products of [IME]^+^Cl^−^ copolymer with all selected reactive dyes were insoluble in DMF, DMSO, methoxypropanol, acetone, pyridine and 1% NaOH. However, the precipitated solid with AB 62 (acid dye) was fully dissolved with the used solvents. These experiments confirmed that coloured pigments were formed in a chemical reaction between reactive dyes and copolymer [IME]^+^Cl^−^ (the covalent bonds were formed). TLC chromatography confirmed the above observation (Figure 11). The Rf values for these products were 0 (remain at the start). The observed Rf values for RR 24:1 and RB 160 may result from hydrolysed original dyes that cannot create a covalent bond with the modification agent.

#### 3.4.3. Density Electron Calculations

**Figure 12:**
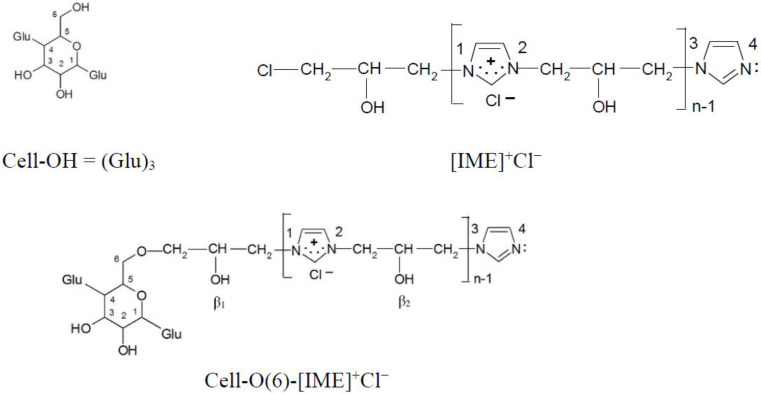
Chemical structure compound models for electron density analysis (ether bond formed with -OH group at C(6) carbon atom).

The dominant direction of epoxide cellulose etherification is attachment to the hydroxyl group located at carbon atom 6, as shown by the earliest calculations of the electron density on all hydroxyl groups in the glucopyranose ring [21]. Such a model (Figure 12) was also selected in this paper for further analysis and calculations.

The formation of [Cell-O(6)-IME]^+^Cl^−^ causes changes in electron densities on the remaining O(2) and O(3) oxygen atoms in the glucopyranose ring (Table 6). The reaction of this product with the reactive dye could proceed according to the following order:

O(β_1_) (−0.319) > O(3) (−0.315) > O(β_2_) (−0.311) > O(2) (−0.286)

However, according to Nishiyama, Langan and Chanzy [27], the hydroxyl groups in positions 2 and 3 in cellulose are very strongly involved in intermolecular hydrogen bonds. The hydroxyl group with the O(β_1_) and O(β_2_) oxygen atom in the [Cell-O(6)-IME]^+^Cl^−^ derivative does not participate in intramolecular hydrogen bonds. They can quickly form a nucleophile and react with the reactive system of the dyes to form a strong covalent bond.

Other calculations of the electron densities carried out on the nitrogen atoms in the modifier chain showed substantial differences, which depend on their location in the chain (Table 7).

Table 7 presents very high electron density differences in nitrogen atoms depending on their location in the cationic modifier chain. The positive charge is dislocated irregularly in the imidazole molecule between N(1) and N(2). Benzenesulfonic acid and acid dye AB 62 were selected to evaluate the strength of the ionic bond between the sulfo group of the reactive dye and the quaternary group in the modified cellulose.

**Figure 13:**
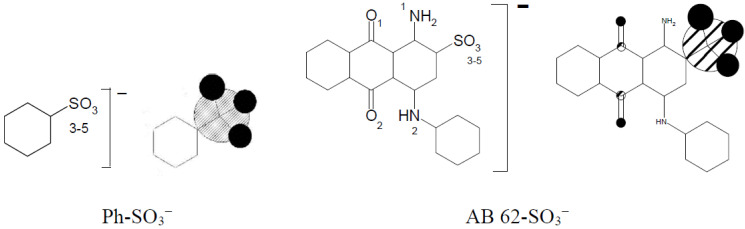
Example of graphical representation of the electron densities on oxygen atoms in sulfo group of benzenenesulfonic acid and AB 62 calculated for their anionic forms.

The total sum of the electron density of all oxygen atoms sulfo groups in Ph-SO_3_^−^ and AB 62-SO_3_^−^ is nearly the same (Table 8) and is −2.820 and −2.812, respectively. It means that using benzenesulfonic acid to form a model compound could be accepted instead of much more complicated dyes with the sulfo group. The formation of an ionic bond between [Cell-O(6)-IME]^+^Cl^−^ and benzenesulfonic acid (Figure 13) causes not only changes in the value of the positive charge on the quaternary group but also changes in the cellulose oxygen atoms in the modifier chain.

**Figure 14:**
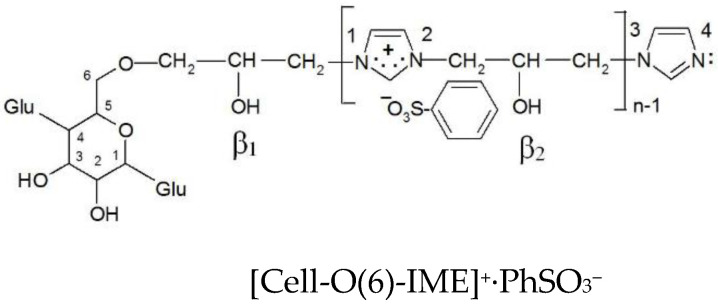
Chemical structure ionic pair cationised cellulose with benzenesulfonic acid for density electron analysis.

Table 9 show that the formation of an ionic bond between the sulfo group and the ammonium group of the modifier reduces the deficit in the cationic imidazole ring (sum of electron density nitrogen atoms from 0.852 in [Cell-O(6)-IME]^+^Cl^−^ to 0.778 in Cell-O(6)-IME]^+^∙PhSO_3_^−^) (Figure 14) and increases the deficit on the adjacent O(β_2_) oxygen atom. The electron density on O(β_2_) in [Cell-O(6)-IME]^+^∙PhSO_3_^−^ is more negative, which makes this hydroxyl group the stronger nucleophile to react with reactive dyes and form covalent bonds in an aqueous bath without the addition of electrolytes and alkali at ambient temperature.

#### 3.4.4. Mixed Dyeing of [Cell-O-IME]^+^Cl^−^ with Reactive and Acid Dyes

**Figure 15:**
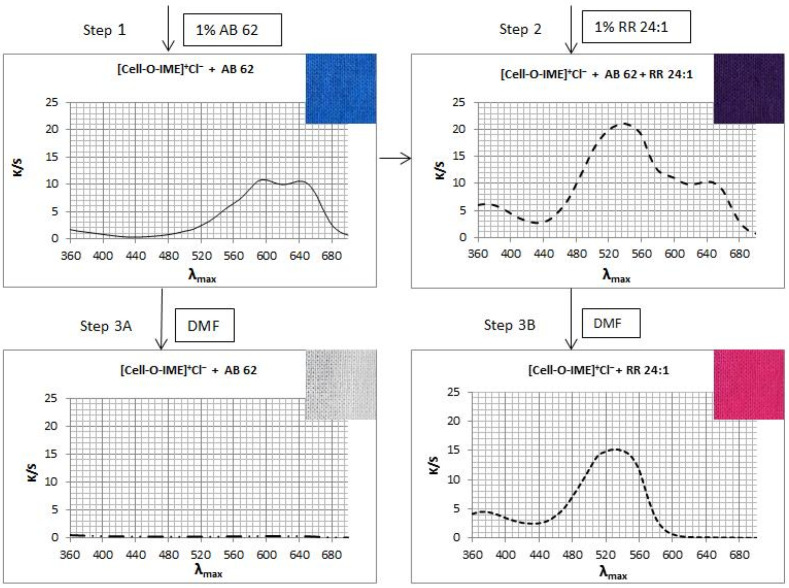
The diagrams presented the running process of mixed dying of [Cell-O-IME]^+^Cl^−^ with RR 24:1 and AB 62 and the extraction results with DMF.

Two samples of [Cell-O-IME]^+^Cl^−^ each ca. 2 g were dyed (Figure 15 step 1) with 1% owf of AB 62 (acid dye) in eco-friendly conditions for 30 min. Then, one sample was removed, and 1% owf of RR 24:1 (reactive dye) was added to the same bath and dyeing was continued (Figure 15 step 2) for 30 min. The bath after dyeing was practically colourless. After dyeing, both samples were rinsed in cold water and dried at room temperature. One half of each of the dyed samples was extracted in boiling DMF (Figure 15 steps 3A and 3B) until the next aliquot was colourless. After extraction, the samples were rinsed in cold water and dried at room temperature. K/S values were measured for all four samples. The following diagram presents the running process:

Mixed dyeing of [Cell-O-IME]^+^Cl^−^ with reactive and acid dyes showed that both dyes under ecological dyeing conditions were exhausted completely and formed ionic bonds with strong ammonium centres. However, being resistant to the extraction treatment with DMF (step 3B), a covalent bond only between [Cell-O-IME]^+^Cl^−^ and RR 24:1 was formed. This was another confirmation of the formation of covalent bonds between the reactive dyes and the hydroxyl/nucleophilic groups in the modifier chain.

## 4. Conclusions

It was found that, during the dyeing of cationised cellulose with a copolymer of [IME]^+^Cl^−^ in a water bath without the addition of electrolytes and alkali at room temperature, reactive dyes of various classes form a covalent bond according to the substitution/addition mechanism with the nucleophile hydroxyl group located in the modifier chain. These bonds were stable for extraction treatment in boiling DMF. TLC chromatography of the insoluble coloured compounds formed in water in the reaction between reactive dyes and the hydroxyl group of the modifier chain confirmed these linkages. Electron densities calculations on oxygen atoms confirmed the experimental results of the high activity of the nucleophile being formed on the hydroxyl group in the modifier chain.

## Figures and Tables

**Table 1:** Information data of the modification agent.

Modification Agent		Chemical Formula
[IME]^+^Cl^−^	1H-imidazole, copolymer with (chloromethyl)oxirane (Texamin ECE New) CAS: 68797-57-9 Molecular weight of 1 mer of copolymer macromolecule [IME]^+^Cl^−^: 160.6	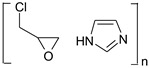 adduct

**Table 2:** Information data of the dyes selected for experiments.

Dyestuff		Chemical Data	Chemical Formula
RR 24:1	Helaktyn Red D-BN Reactive Red 24:1 C.I. 18208:1 CAS: 72829-25-5	MCT Molecular Formula C_27_H_19_ClN_7_O_10_S_3_.Na_3_ Molecular weight: 802.10	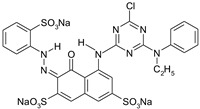
RB 160	Kalpactive Blue HE-BR Reactive Blue 160 C.I. - CAS: 71872-76-9	bis-MCT formazan class dyes Molecular Formula C_38_H_23_Cl_2_N_14_O_18_S_5_Cu.Na_5_ Molecular Weight: 1309.9	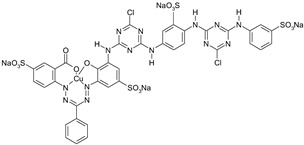
RR 221	Papizolon Red HT-3BN Reactive Red 221 C.I. - CAS: 96726-27-1	bis-mononicotinntriazine dyes Molecular Formula C_57_H_35_N_16_O_24_S_6_.Na_6_ Molecular Weight: 1699.34	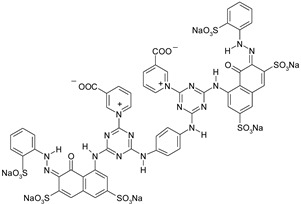
RR 274	Eriofast Red 2B ^(1)^ Reactive Red 274 C.I. - CAS: -	MCT-VS (heterobifunctional) Molecular Formula C_27_H_20_N_8_O_9_S_3_Cl.Na_2_ Molecular Weight: 777.5	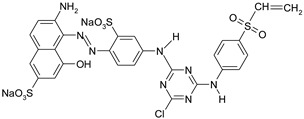
RB 19	Remazol Brilliant Blue R Reactive Blue 19 C.I. 61200 CAS: 2580-78-1	VS (vinylsulphone) Molecular Formula C_22_H_16_N_2_O_11_S_3_.Na_2_ Molecular Weight 626.5	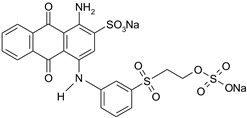
AB 62	Acid Blue 62 C.I. 62045 CAS: 4368-56-3	Molecular Formula C_20_H_20_N_2_O_5_S.Na Molecular Weight 422.43	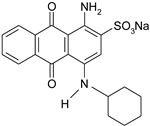

^(1)^ Chemical formula known from [22].

**Table 3:** Summary of data for [IME]^+^Cl^−^copolymer.

Nitrogen Content	$Q_{{\left[IME\right]}^{+}Cl^{-}}^{+}$	$R_{{\left[IME\right]}^{+}Cl^{-}\;}$
[eq/g]		
5.16 × 10^−3^	2.1 × 10^−3^	0.515 × 10^−3^

**Table 4:** Calculated values of nitrogen content and specific surface charge for uncationised and cationised cotton samples.

Cotton Sample	Nitrogen		$Q_{surf}^{+}\;$	
	Calculated	Δ*N*	Calculated	$\Delta Q_{surf}^{+}\;$
	[10^−6^ eq/g]		[10^−6^ eq/g]	
Cell-OH	25	-	+0.44	-
[Cell-O-IME]^+^Cl^−^	126	101	+14.2	+13.76

**Table 5:** Resistance data L a b and DMF extraction for dyed samples of [Cell-O-IME]^+^Cl^−^ and Cell-OH.

Dye	Dyeing in Eco-Friendly Conditions													
	Cell-OH							Cell-O-[IME]^+^Cl^−^						
	After Dyeing			After DMF Extraction			*R_DMF_* [%]	After Dyeing			After DMF Extraction			*R_DMF_* [%]
	L	a	b	L	a	b		L	a	b	L	a	b	
RR 24:1	80.18	25.28	−2.40	92.39	2.71	1.82	8.10	46.40	64.93	13.46	43.11	61.22	7.81	92.47
	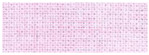			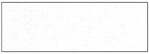				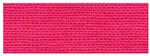			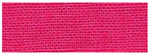			
RB 160	59.81	−5.30	−22.07	76.98	−3.83	−12.33	21.1	43.85	−3.77	−27.92	44.20	−3.03	−27.78	94.90
	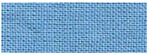			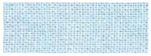				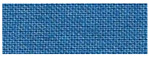			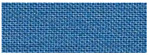			
RR 221	64.26	43.92	−4.07	74.97	29.49	−3.27	33.9	45.86	57.29	3.53	46.25	57.35	3.18	96.37
	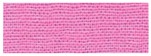			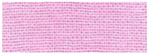				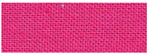			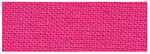			
RR 274	57.13	42.15	−6.10	82.06	15.90	−3.87	8.80	42.15	50.99	−1.09	40.63	52.62	−1.15	103.3
	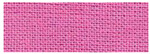			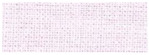				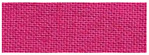			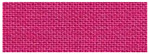			
RB 19	60.30	−6.06	−14.98	86.24	−1.88	−2.97	6.85	31.13	−5.06	−20.03	31.41	−5.22	−21.17	98.30
	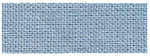			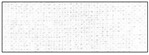				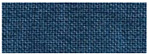			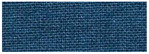			

**Table 6:** Calculated electron-density data for analysed oxygen atoms.

Compound	Electron Density on An Oxygen Atoms				
	O(6)	O(2)	O(3)	O(β_1_)	O(β_2_)
[IME]^+^Cl^−^	---	---	---	−0.325	−0.315
Cell-OH = (Glu)_3_	−0.341	−0.287	−0.291	---	---
[Cell-O(6)-IME]^+^Cl^−^	−0.275	−0.286	−0.315	−0.319	−0.311

**Table 7:** Electron densities on the selected atoms of [Cell-O(6)-IME]^+^Cl^−^.

Atom	Electron Density
N(1)	0.445
N(2)	0.407
N(3)	0.236
N(4)	−0.085

**Table 8:** The summary data of electron densities of oxygen atoms in the sufo group of benzenesulfonic acid and AB 62.

Compound	O(3)	O(4)	O(5)
Ph-SO_3_^−^	−0.939	−0.939	−0.942
AB 62-SO_3_^−^	−0.956	−0.916	−0.940

**Table 9:** The summary data electron density for analysed oxygen atoms of [Cell-O(6)-IME]^+^∙PhSO_3_^−^.

Compound	Electron Density on Oxygen and Nitrogen Atoms					
	O(2)	O(3)	O(β_1_)	O(β_2_)	N(1)	N(2)
[Cell-O(6)-IME]^+^Cl^−^	−0.286	−0.315	−0.319	−0.311	0.445	0.407
[Cell-O(6)-IME]^+^∙PhSO_3_^−^	−0.292	−0.311	−0.311	−0.318	0.410	0.368

## Data Availability

Not applicable.

## Abbreviations

AB 62	Nylanthrene Brilliant Blue 2RFF (Acid Blue 62)
Cell-OH	cellulose
[CHPTA]^+^Cl^−^	3-chloro-2-hydroxymethyltrimethylammonium chloride (CHPTAC)
[Cell-O-IME]^+^Cl^−^	cellulose cationised with copolymer (chloromethyl)oxirane-1H-imidazole
DMF	N,N-dimethylformamide
DMSO	dimethyl sulfoxide
Glu	glucopiranose ring
[IME]^+^Cl^−^)	copolymer (chloromethyl)oxirane-1H-imidazole
Kayacelon React	brand name monochlorotriazine type of reactive dyes with nicotinic group
LR	liquor ratio
MCT	monochlorotriazine type of reactive dyes
owf	on weight fibre
PhSO_3_H	benzenesulfonic acid
PES-Na	polystyrene sulfonic acid natrium salt
poly-DADMAC	polydiallyldimethylammonium chloride
Poly(St-BA-VBT)	poly(styrene-butyl acrylate-vinylbenzyl trimethylammonium chloride)
RB 19	Remazol Brilliant Blue R (Reactive Blue 19)
RB 160	Kalpactive Blue HE-BR (Reactive Blue 160)
RR 24:1	Helaktyn Red D-BN (Reactive Red 24:1)
RR 221	Papizolon Red HT-3BN (Reactive Red 221)
RR 274	Eriofast Red 2B (Reactive Red 274)
TLC	thin layer chromatography
VS	vinylosulfone type of reactive dyes
